# Knowledge, Attitudes, and Preventive Practices Regarding Thyroid Disorders During Pregnancy: A Nationwide Online Cross-Sectional Study: Short title: Thyroid Knowledge and Practices in Pregnancy

**DOI:** 10.31661/gmj.v15i.4154

**Published:** 2026-09-29

**Authors:** Basil Mohammed Alomair

**Affiliations:** ^1^ Internal Medicine Department, College of Medicine, Jouf University, Saudi Arabia

**Keywords:** Thyroid Disorders, Pregnancy, Hypothyroidism, Hyperthyroidism, Knowledge, Attitudes, Saudi Arabia

## Abstract

**Background:**

Thyroid dysfunction during pregnancy is an important endocrine concern because untreated disease may adversely affect maternal health, pregnancy outcomes, and fetal neurodevelopment. Public awareness is particularly relevant in Saudi Arabia, where historical iodine deficiency, regional variation in dietary iodine intake, and differences in health literacy may influence preventive behaviors. This study assessed knowledge, attitudes, and preventive practices regarding thyroid disorders during pregnancy among Saudi adults of reproductive age.

**Materials and Methods:**

A nationwide online cross-sectional survey was conducted among 1,053 Saudi adults aged 18-45 years from the five major geographical regions of Saudi Arabia. Participants completed a structured self-administered questionnaire assessing sociodemographic characteristics, knowledge of thyroid disorders in pregnancy, attitudes toward screening and treatment, preventive practices, and sources of health information. Knowledge, attitude, and practice scores were summarized using descriptive statistics. Group differences were examined using independent-samples t-tests or one-way analysis of variance, as appropriate.

**Results:**

The mean knowledge score was moderate at 5.9 ± 2.4 out of 10. Although most participants recognized that thyroid disorders can be diagnosed using blood tests (84.2%) and may affect fetal brain development (76.1%), only 52.1% knew that thyroid disorders during pregnancy may be asymptomatic. Attitudes were generally favorable, with a mean score of 26.9 ± 4.5 out of 35; 82.9% agreed or strongly agreed that thyroid screening is important in prenatal care, and 96.0% recognized the importance of management for fetal health. Preventive practices were less optimal, with a mean score of 5.4 ± 2.3 out of 10. Only 44.1% had previously undergone thyroid function testing, and 30.1% regularly consumed iodine-rich foods.

**Conclusion:**

Saudi adults of reproductive age demonstrated favorable attitudes but only moderate knowledge and suboptimal preventive practices regarding thyroid disorders during pregnancy. These findings support targeted public health education, improved preconception counseling, and clearer clinical communication regarding thyroid screening and treatment safety during pregnancy.

## Introduction

Pregnancy is accompanied by substantial endocrine and metabolic adaptations that increase maternal thyroid hormone requirements [1, 2]. These physiological changes are driven partly by increased thyroxine-binding globulin concentrations, stimulation of the thyroid gland by human chorionic gonadotropin, and increased iodine requirements [1, 3]. Adequate maternal thyroid hormone availability is essential during early gestation because fetal neurodevelopment depends on maternal thyroxine before maturation of the fetal thyroid axis [4, 5, 6].

Thyroid dysfunction is among the most clinically relevant endocrine disorders encountered during pregnancy [2, 7]. Overt and subclinical hypothyroidism, as well as hyperthyroidism, may complicate pregnancy and require timely recognition, pregnancy-specific interpretation of thyroid function tests, and individualized management [7, 8]. Contemporary clinical guidance emphasizes optimization of thyroid care before conception, during pregnancy, and postpartum because delayed diagnosis, undertreatment, and overtreatment may all carry maternal or fetal consequences [7, 9].

Untreated hypothyroidism during pregnancy has been associated with adverse maternal and fetal outcomes, including hypertensive disorders of pregnancy, miscarriage, preterm birth, impaired fetal growth, and adverse neurodevelopmental outcomes [10, 11, 12]. Uncontrolled hyperthyroidism may increase the risk of pregnancy loss, preeclampsia, preterm delivery, fetal growth restriction, and maternal complications [7, 13]. Although risk varies according to disease severity, antibody status, iodine sufficiency, and timing of treatment, early recognition remains an important component of maternal health care [7, 9].

Saudi Arabia provides an important setting for evaluating public awareness of thyroid disorders during pregnancy [14, 15]. Historical iodine deficiency, variation in dietary iodine intake, and differences in access to reliable health information may influence the population’s understanding of thyroid health [15, 16]. Iodine remains essential for synthesis of thyroxine and triiodothyronine, and pregnancy increases iodine requirements because of increased thyroid hormone production and fetal needs [3, 15]. Despite the clinical relevance of thyroid dysfunction in pregnancy, public knowledge and preventive behavior remain insufficiently characterized in Saudi Arabia [14, 17]. Existing regional studies are limited by sample size, local recruitment, or focus on thyroid disease awareness outside the pregnancy context [14]. Understanding knowledge, attitudes, and practices among adults of reproductive age may help identify modifiable gaps in awareness, screening behavior, and preconception counseling [17].

Therefore, this study aimed to assess knowledge, attitudes, and preventive practices regarding thyroid disorders during pregnancy among Saudi adults aged 18-45 years across the major geographical regions of Saudi Arabia.

## Materials and Methods

### Study Design

This nationwide online cross-sectional survey was designed to assess knowledge, attitudes, and preventive practices regarding thyroid disorders during pregnancy among Saudi adults of reproductive age.

### Study Setting and Population

The study targeted Saudi citizens aged 18-45 years residing in the five major geographical regions of Saudi Arabia: Central, Western, Eastern, Southern, and Northern regions. Eligible participants were Saudi adults within the specified age range who were able to complete the online questionnaire and provided electronic informed consent. Participants who reported a previous diagnosis of thyroid disease were excluded to focus on baseline public knowledge and preventive practices among individuals without direct personal experience of thyroid disease. This criterion may reduce generalizability to the broader population and is therefore addressed as a study limitation.

### Sampling Technique and Sample Size

Participants were recruited using a non-probability online sampling strategy with regional targeting to improve geographical representation. The survey link was distributed through social media platforms, university and workplace mailing lists, community groups, and health-related digital networks. The minimum required sample size was calculated using the single-proportion formula, assuming a 50% prevalence of adequate knowledge, a 95% confidence level, and a 5% margin of error. After accounting for potential incomplete responses and the need for regional representation, the target sample size was increased to approximately 1,000 participants. A total of 1,053 complete responses were included in the final analysis.

### Data Collection Tool

Data were collected using a structured, self-administered Arabic questionnaire developed after reviewing relevant literature on thyroid disorders during pregnancy and knowledge, attitude, and practice survey methodology. The questionnaire assessed sociodemographic characteristics, knowledge regarding thyroid disorders during pregnancy, attitudes toward thyroid screening and treatment, preventive practices related to thyroid health, and primary sources of health information. The questionnaire included five sections: sociodemographic characteristics; knowledge regarding thyroid disorders during pregnancy; attitudes toward thyroid screening, treatment, and follow-up; preventive practices related to thyroid health; and primary sources of health information. The knowledge domain included 10 items, with one point assigned for each correct answer and zero points assigned for incorrect or “I do not know” responses. Total knowledge scores ranged from 0 to 10 and were categorized as poor, average, or good. The attitude domain included seven items assessed using a five-point Likert scale ranging from “strongly disagree” to “strongly agree.” Negatively worded items were reverse-scored where appropriate, and total attitude scores ranged from 7 to 35, with higher scores indicating more favorable attitudes. The practice domain included five items assessing previous thyroid testing, intention to seek preconception thyroid screening, physician recommendation of thyroid testing during previous pregnancy, iodine-rich food consumption, and willingness to adhere to prescribed thyroid medication. Total practice scores ranged from 0 to 10, with higher scores indicating more proactive preventive practices. Although the questionnaire was developed based on published literature and KAP survey methodology, formal psychometric validation and reliability testing were not completed before data collection; this limitation should be considered when interpreting the KAP scores.

### Ethical Approval

The study was approved by the Biomedical Research Ethics Committee (approval number: HAPO-02-K-012-2025-11-3002). Participation was voluntary, and electronic informed consent was obtained before participants accessed the questionnaire. The first page of the survey explained the study objectives, confidentiality procedures, anonymity of responses, and the right to withdraw before submission. No personally identifiable information was collected. The study was conducted in accordance with the ethical principles of the Declaration of Helsinki.

### Statistical Analysis

Data were exported from the online survey platform, screened for completeness, coded, and analyzed using statistical software. Categorical variables were summarized as frequencies and percentages. Continuous variables, including knowledge, attitude, and practice scores, were summarized as means and standard deviations. Independent-samples t-tests were used to compare mean KAP scores between two-group variables, including gender and pregnancy history. One-way analysis of variance was used to compare mean scores across variables with more than two categories, including age group, region, educational level, and employment status. A two-sided P<0.05 was considered statistically significant. The analysis was limited to bivariate comparisons. Multivariable regression should be considered in future analyses to identify independent predictors of knowledge, attitude, and practice scores.

## Results

**Table:** Table1. Sociodemographic Characteristics of the Study Participants

Variable	Category	N	%
	18–24	316	30.0
Age group, years	25–34	421	40.0
	35–45	316	30.0
Gender	Female	901	85.6
	Male	152	14.4
	Central	295	28.0
	Western	274	26.0
Region of residence	Eastern	221	21.0
	Southern	168	16.0
	Northern	95	9.0
	Single	337	32.0
Marital status	Married	685	65.1
	Divorced/Widowed	31	2.9
	High school or below	211	20.0
Educational level	Diploma	274	26.0
	Bachelor’s degree	443	42.1
	Postgraduate degree	125	11.9
Employment status	Student	232	22.0
	Unemployed	126	12.0
	Employed	422	40.1
	Homemaker	273	25.9
Pregnancy history	Never pregnant	401	38.1
	Ever pregnant	652	61.9
Current pregnancy status among ever-pregnant participants	Currently pregnant	148	22.7

**Note:** Percentages were calculated using the total sample size unless otherwise indicated. Current pregnancy status was calculated among participants with a history of pregnancy only.

**Table:** Table2. Knowledge Regarding Thyroid Disorders During Pregnancy

Knowledge item / classification	Correct response N(%)	Incorrect response N(%)	“I do not know” N(%)
The thyroid gland is part of the endocrine system	844 (80.2)	126 (12.0)	83 (7.9)
Thyroid hormone levels remain unchanged during pregnancy*	633 (60.1)	221 (21.0)	199 (18.9)
Correctly identified symptoms of hypothyroidism	695 (66.0)	238 (22.6)	120 (11.4)
Correctly identified symptoms of hyperthyroidism	654 (62.1)	275 (26.1)	124 (11.8)
Untreated thyroid disorders increase the risk of miscarriage/pre-eclampsia	759 (72.1)	158 (15.0)	136 (12.9)
Untreated thyroid disorders affect the baby’s brain development	801 (76.1)	126 (12.0)	126 (12.0)
Thyroid disorders during pregnancy are always symptomatic*	548 (52.1)	316 (30.0)	189 (17.9)
Thyroid disorders can be diagnosed by a blood test	887 (84.2)	95 (9.0)	71 (6.7)
Thyroid medication is generally safe during pregnancy	717 (68.1)	211 (20.0)	125 (11.9)
Adequate iodine intake is important for thyroid health during pregnancy	845 (80.2)	116 (11.0)	92 (8.7)
			
Knowledge score classification	Score range	N(%)	
Poor knowledge	0–4	295 (28.0)	
Average knowledge	5–7	506 (48.0)	
Good knowledge	8–10	252 (23.9)	
Mean knowledge score (SD) = 5.9 (2.4)			

**Note:** Each correct answer was scored as 1 point; incorrect and “I do not know” responses were scored as 0. Items marked with * were negatively phrased; the correct response required identifying the statement as false.

**Table:** Table3. Attitudes Toward Thyroid Screening, Treatment, and Follow-Up During Pregnancy

Attitude statement / classification	Strongly disagree N(%)	Disagree N(%)	Neutral N(%)	Agree N(%)	Strongly agree N(%)
Thyroid screening is important during prenatal care	21 (2.0)	32 (3.0)	127 (12.1)	422 (40.1)	451 (42.8)
I am worried about potential side effects of thyroid medication during pregnancy*	95 (9.0)	211 (20.0)	306 (29.1)	317 (30.1)	124 (11.8)
If diagnosed, I would be committed to regular medical follow-up	11 (1.0)	21 (2.0)	84 (8.0)	443 (42.1)	494 (46.9)
Thyroid problems during pregnancy are rare and not a major concern*	337 (32.0)	422 (40.1)	190 (18.0)	84 (8.0)	20 (1.9)
Managing thyroid disease is crucial for fetal health	0 (0.0)	0 (0.0)	42 (4.0)	464 (44.1)	547 (51.9)
The cost of thyroid testing is a barrier for me*	253 (24.0)	359 (34.1)	211 (20.0)	190 (18.0)	40 (3.8)
I feel confident in my knowledge about thyroid health during pregnancy	169 (16.1)	306 (29.1)	359 (34.1)	190 (18.0)	29 (2.8)
					
Attitude score classification	Score range	N(%)			
Negative attitude	7–20	147 (14.0)			
Neutral attitude	21–27	422 (40.1)			
Positive attitude	28–35	484 (46.0)			
Mean attitude score (SD) = 26.9 (4.5)					

**Note:** Items marked with * were negatively worded and reverse-scored.

**Table:** Table4. Practices Regarding Thyroid Health Before and During Pregnancy

Practice item / classification	Response / score range	N	%
Ever had thyroid function tested	Yes	464	44.1
	No	506	48.1
	I do not know / cannot remember	83	7.9
Would seek preconception counseling including thyroid assessment	Yes, definitely	549	52.2
	Maybe	338	32.1
	No / Not applicable	166	15.8
Doctor recommended or performed thyroid testing during a previous pregnancy	Yes	380	58.3
	No	127	19.5
	I do not remember	145	22.2
Consumes iodine-rich foods	Yes, regularly	317	30.1
	Sometimes	506	48.1
	Rarely/Never	230	21.8
Would take prescribed thyroid medication as directed if diagnosed during pregnancy	Very likely	759	72.1
	Somewhat likely	211	20.0
	Neutral/Unlikely/Not applicable	83	7.9
			
Practice score classification	Score range	N(%)	
Poor preventive practices	0–4	359 (34.1)	
Moderate preventive practices	5–7	506 (48.1)	
Good preventive practices	8–10	188 (17.9)	
Mean practice score (SD) = 5.4 (2.3)			

**Note:** For “Doctor recommended or performed thyroid testing during a previous pregnancy,” percentages were calculated among participants with a history of pregnancy only (n=652). Higher scores indicate more proactive preventive practices.

**Figure-1:**
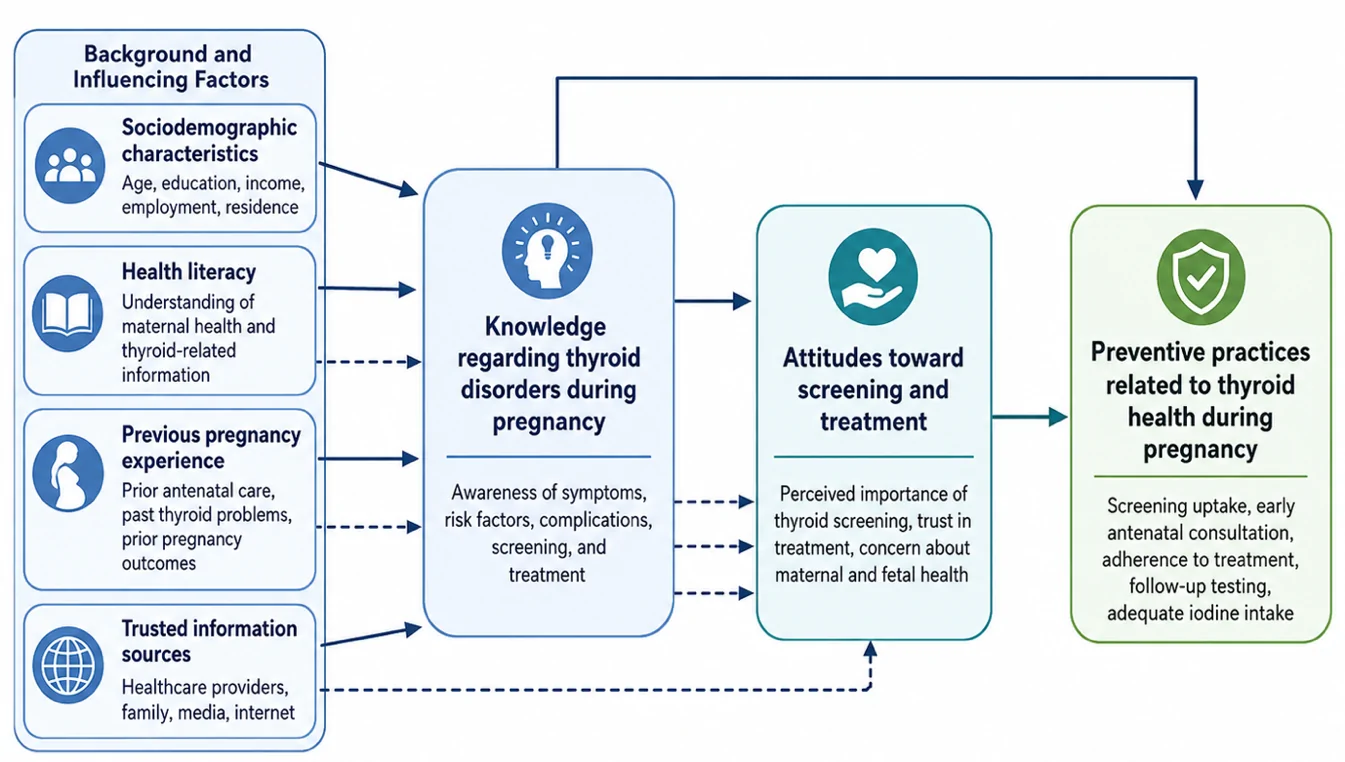
Figure 1. Conceptual Framework Linking Sociodemographic Characteristics, Knowledge, Attitudes, and Preventive Practices Regarding Thyroid Disorders During Pregnancy

### Participant Characteristics

A total of 1,053 participants were included in the analysis. Most respondents were female (85.6%), and the largest age group was 25-34 years (40.0%). Participants were recruited from all five major geographical regions of Saudi Arabia, with the highest representation from the Central region (28.0%), followed by the Western region (26.0%). Most participants were married (65.1%), and 54.0% had a bachelor’s or postgraduate degree. A history of pregnancy was reported by 61.9% of participants; among those with previous pregnancy experience, 22.7% were currently pregnant (Table1).

### Knowledge Regarding Thyroid Disorders During Pregnancy

The mean knowledge score was 5.9 ± 2.4 out of 10, indicating a moderate level of knowledge. Overall, 28.0% of participants had poor knowledge, 48.0% had average knowledge, and 23.9% had good knowledge. Most participants correctly identified that the thyroid gland is part of the endocrine system (80.2%), that thyroid disorders can be diagnosed by blood testing (84.2%), and that untreated thyroid disorders may affect fetal brain development (76.1%). However, important knowledge gaps were observed. Only 52.1% recognized that thyroid disorders during pregnancy may be asymptomatic, and 60.1% correctly understood that thyroid hormone levels do not remain completely unchanged during pregnancy (Table2).

### Attitudes Toward Thyroid Health During Pregnancy

The mean attitude score was 26.9 ± 4.5 out of 35. Nearly half of the participants had a positive attitude score, while 40.1% had a neutral attitude score. Most participants agreed or strongly agreed that thyroid screening is important during prenatal care (82.9%) and that management of thyroid disease is crucial for fetal health (96.0%) (Table3). Despite these favorable attitudes, 41.9% of participants expressed concern about the potential side effects of thyroid medication during pregnancy. In addition, only 20.8% agreed or strongly agreed that they felt confident in their knowledge about thyroid health during pregnancy (Table3).

### Preventive Practices

The mean practice score was 5.4 ± 2.3 out of 10. Preventive practices were generally suboptimal: 34.1% of participants had poor practice scores, 48.1% had moderate scores, and only 17.9% had good scores. Less than half of participants had previously undergone thyroid function testing (44.1%). Among participants with a history of pregnancy, 58.3% reported that a physician had recommended or performed thyroid testing during a previous pregnancy. Although 52.2% stated that they would definitely seek preconception counseling including thyroid assessment, only 30.1% reported regular consumption of iodine-rich foods (Table4).

### Sources of Health Information

Healthcare professionals were the most commonly reported primary source of pregnancy-related health information (44.1%), followed by internet websites and search engines (30.0%) and social media platforms (14.1%). This finding suggests that both clinical counseling and digital health communication may be important channels for improving thyroid-related awareness.

### Sociodemographic Factors Associated with KAP Scores

Knowledge, attitude, and practice scores varied across sociodemographic groups. Higher knowledge and practice scores were generally observed among older participants, women, married participants, those with higher educational attainment, employed participants, and participants with previous pregnancy experience.

Knowledge, attitude, and practice scores varied according to several sociodemographic characteristics (Figure-1). Higher mean scores were generally observed among older participants, women, participants with higher educational attainment, employed participants, and those with a history of pregnancy. Region of residence was significantly associated with knowledge score but was not significantly associated with attitude or practice scores based on the reported P-values.

## Discussion

This nationwide online survey identified moderate knowledge, generally favorable attitudes, and suboptimal preventive practices regarding thyroid disorders during pregnancy among Saudi adults of reproductive age [14, 17]. The findings indicate a gap between recognition of the importance of thyroid health and engagement in preventive behaviors such as thyroid function testing, preconception counseling, and regular iodine-related dietary practices [15, 17].

A key finding was that participants showed only moderate knowledge despite recognizing several major clinical concepts [14, 17]. Most respondents knew that thyroid disorders can be diagnosed by blood testing and that untreated disease may affect fetal brain development [4, 5, 14]. However, only approximately half recognized that thyroid disorders during pregnancy may be asymptomatic [7, 8].

This gap is clinically important because subclinical thyroid dysfunction may not produce obvious symptoms, and reliance on symptoms alone may delay testing or diagnosis [7, 8].

The observed pattern is consistent with recent international KAP research. A cross-sectional study from Guangdong, China, similarly reported insufficient knowledge and inactive practices regarding thyroid disorders among pregnant women despite generally positive attitudes [18]. Together, these findings suggest that thyroid-related pregnancy education remains inadequate across different health systems and cultural settings [14, 18].

The contrast between favorable attitudes and weaker practices is one of the most important findings of this study [14, 17]. Most participants agreed that thyroid screening is important during prenatal care and that disease management is important for fetal health [7, 14]. Nevertheless, fewer than half had ever undergone thyroid function testing, and only about one-third regularly consumed iodine-rich foods [15, 17]. This indicates that positive attitudes alone may not translate into preventive behavior unless accompanied by practical access, clear clinical recommendations, and reliable health information [14, 17, 18].

Medication-related concern was another important barrier. More than two-fifths of respondents expressed worry about thyroid medication side effects during pregnancy [14]. This concern is clinically relevant because fear of medication may reduce adherence if thyroid dysfunction is diagnosed [7, 9]. Patient counseling should therefore explain the relative risks of untreated thyroid disease and the role of indicated therapy [7, 10]. Current reviews and guidance emphasize that levothyroxine remains the standard treatment for overt hypothyroidism in pregnancy and that treatment decisions for subclinical disease should consider thyroid-stimulating hormone level, thyroid antibody status, gestational timing, and guideline-specific thresholds [7, 9, 19].

The findings also have implications for public health communication in Saudi Arabia [14, 15]. Healthcare professionals were the most trusted information source, but nearly one-third of participants relied primarily on internet sources and a substantial minority relied on social media [14, 17]. This pattern supports a dual strategy: strengthening clinician-led counseling during preconception and antenatal care while also developing evidence-based Arabic digital educational materials [14, 17].

Sociodemographic differences in KAP scores suggest that educational interventions should prioritize younger adults, men, participants with lower educational attainment, and individuals without previous pregnancy experience [14, 17]. Although women are directly affected by pregnancy-related thyroid disorders, men may influence family health decisions and support care-seeking behavior [17]. Therefore, public health campaigns should not exclude men [17].

The Saudi context makes these findings particularly relevant [15, 16]. Iodine is essential for thyroid hormone synthesis, and pregnancy increases iodine requirements [3, 15]. Public education should therefore include accurate information on iodine sources, iodized salt, and appropriate supplementation where clinically indicated [15, 16].

This study has several strengths. It included a large sample from all major regions of Saudi Arabia and addressed knowledge, attitudes, practices, and information sources in a single survey [14, 17]. The findings provide a useful baseline for future educational interventions and policy planning [14].

Overall, the study demonstrates a meaningful public health gap: favorable attitudes toward thyroid health in pregnancy are not matched by adequate knowledge or preventive practices. Addressing this gap will require integrated educational interventions, improved preconception counseling, and consistent clinical messaging regarding thyroid screening and treatment safety during pregnancy.

## Limitations

This study has several limitations. First, its cross-sectional design does not allow causal inference. Second, the online non-probability recruitment strategy may have introduced selection bias by favoring participants with internet access, higher educational attainment, or greater interest in health-related topics. Third, all practices were self-reported and may therefore be affected by recall bias or social desirability bias. Fourth, the questionnaire was developed based on literature review, but formal psychometric validation, expert-panel content validity assessment, pilot testing, translation/back-translation, and reliability testing were not completed before data collection; this may affect the validity, reliability, and interpretability of the KAP scores. Fifth, some pregnancy-specific practice items may not apply equally to all respondents, particularly men and participants without previous pregnancy experience. Finally, the analysis was limited to bivariate comparisons; multivariable regression analysis is needed to identify independent predictors of knowledge, attitudes, and practices.

## Conclusion

Saudi adults of reproductive age demonstrated favorable attitudes but only moderate knowledge and suboptimal preventive practices regarding thyroid disorders during pregnancy. Important gaps were observed in awareness of asymptomatic thyroid disease, previous thyroid testing, and regular iodine-related dietary practices. These findings highlight the need for targeted public health education, clinician-led counseling, and reliable Arabic digital health resources to improve thyroid-related awareness before and during pregnancy. Future studies should use validated instruments, probability-based sampling where feasible, and multivariable analysis to identify independent determinants of preventive thyroid health behaviors.

## Conflict of Interest

The author declares that there are no conflicts of interest regarding the publication of this study.

## AI Disclosure Statement

The author declares that artificial intelligence-assisted tools (ChatGPT) were used only for language editing, grammar correction, and formatting support during manuscript preparation. Scientific figures were created using FigureLabs, an AI-assisted scientific illustration tool, based on concepts developed by the authors. The author reviewed and approved the final manuscript and takes full responsibility for the scientific content.
